# Acceptability and exploratory effects of an occupational therapy intervention to improve recovery and return to work of workers with mental health disorders in primary care: a mixed methods study protocol

**DOI:** 10.3389/fpsyt.2024.1441855

**Published:** 2024-11-28

**Authors:** Justine Labourot, Quan Nha Hong, Catherine Briand, Cynthia Cameron, Marie-José Durand, Nadia Giguère, Élyse Marois, Matthew Menear, Marielle Trottier, Helen-Maria Vasiliadis, Brigitte Vachon

**Affiliations:** ^1^ École de réadaptation, Faculté de médecine, Université de Montréal, Montréal, QC, Canada; ^2^ Centre de Recherche de l’Institut Universitaire en Santé Mentale de Montréal, Montréal, QC, Canada; ^3^ Centre for Interdisciplinary Research in Rehabilitation of Greater Montreal (CRIR), Montréal, QC, Canada; ^4^ Département d'ergothérapie, Université du Québec à Trois-Rivières, Trois-Rivières, QC, Canada; ^5^ Department of Family Medicine and Emergency Medicine, Faculty of Medicine, Université Laval, Québec City, QC, Canada; ^6^ École de Réadaptation, Faculté de Médecine et des Sciences de la Santé, Université de Sherbrooke, Sherbrooke, QC, Canada; ^7^ Centre d’action en Prévention et Réadaptation de l’incapacité au Travail, Longueuil, QC, Canada; ^8^ Department of Family Medicine and Emergency Medicine, Faculty of Medicine, Université de Montréal, Montréal, QC, Canada; ^9^ Centre de recherche de Montréal sur les Inégalités sociales, les discriminations et les pratiques alternatives de citoyenneté (CREMIS), Montréal, QC, Canada; ^10^ VITAM Research Centre for Sustainable Health, Québec, QC, Canada; ^11^ University of Montreal Hospital Centre (CRCHUM), Montréal, QC, Canada; ^12^ Department of Community Health Sciences, Faculty of Medicine and Health Sciences, Université de Sherbrooke, Sherbrooke, QC, Canada; ^13^ Charles-Le Moyne Research Center, Longueuil, QC, Canada

**Keywords:** primary care, common mental disorders, occupational therapy, return to work coordination, acceptability, implementation

## Abstract

**Background:**

People with common mental disorders (CMD) are prone to experience work disabilities, which can lead to sick leave. To support their recovery and return to work, evidence recommends providing a combination of primary care services including psychological and work rehabilitation interventions. Furthermore, interventions to coordinate return to work are required to ensure timely access to services and concerted action among stakeholders. Occupational therapists are qualified to provide these interventions and to facilitate sick leave management. However, current medical practices, lack of collaboration among stakeholders, and lack of occupational therapists working within family medicine groups create highly variable care pathways and delays in access to appropriate services.

**Aim:**

This study aims to evaluate the acceptability and explore the effects of an occupational therapist-led program integrated within family medicine groups designed to improve the management of CMD-related sick leave and promote patients' recovery and sustainable return to work in the Canadian province of Québec.

**Methods:**

This study will consist of a mixed methods multiple case study design. It will also use a participatory research approach, actively engaging family medicine group team members and patient partners throughout the study. The occupational therapy program will include three components: 1) consultation for prevention of sick leave and support for return-to-work decisions, 2) coordination of recovery and return-to-work services, and 3) provision of recovery and work rehabilitation services adapted to each patient’s needs. Questionnaires, interviews, and focus groups will be used to collect data on the eight dimensions of the acceptability model described by Sekhon et al. and to measure pre- and post-outcomes to assess the effects of the occupational therapy program. Data will be analyzed using the Framework Method and repeated measures statistical analysis.

**Discussion:**

We expect that the provision of this innovative occupational therapy program will improve patients’ outcomes and the service trajectory of people with CMD. This study will document how to enhance interprofessional collaboration within family medicine groups and to ensure equitable access to work rehabilitation services for all patients, thereby improving recovery and healthy sustainable return-to-work.

## 1 Introduction

Approximately half of the world’s population will experience a mental disorder affecting their day-to-day functioning and quality of life during their lifetime (1). These disorders affect work capacity and are leading causes of presenteeism, absenteeism, and staff turnover (2). This situation contributes to significant societal and economic costs worldwide. For example, in Canada, half a million workers will be absent from work each week due to a mental disorder (3) and 20% of those with a common mental disorder (CMD) will experience prolonged work disability (4, 5). People experiencing prolonged sick leave are at greater risk of relapse and present reduced chances of recovery and return-to-work (RTW) (6). In Canada, it is estimated that mental disorders incur an annual cost of over $50 billion, of which between $6 billion and $19 billion can be attributed to lost productivity related to absenteeism, presenteeism, and permanent withdrawal from the job market (7).

In this context, the need for efficient and accessible mental health services is undeniable (8). Yet, according to the World Health Organization, mental health systems and services around the world remain inaccessible or poorly adapted to meet these needs (8). This problem persists despite clear evidence on sick leave management for people with CMDs. Evidence shows that people who are on CMD-related sick leave benefit from interventions provided by an interdisciplinary team. These interventions should combine pharmaceutical, psychological, and work rehabilitation interventions to improve their recovery and their healthy and sustainable RTW (2, 9–14). Clinical approaches, such as cognitive-behavioral therapy, should be combined with work-focused interventions, such as work tasks and work environment modifications, as psychological interventions alone were found to be insufficient to support a healthy and sustainable RTW (2, 15). These interventions should be offered to the individual promptly and in collaboration with the stakeholders involved which includes but is not limited to the patient, the healthcare professionals, the employer, and, when appropriate, the insurer (2, 11, 16–19). Furthermore, these interventions should be delivered within a recovery-oriented approach to care to ensure that patients’ goals, choices, and strengths are respected and promoted throughout their recovery and RTW process (8, 20). Ultimately, to optimize the support provided during sick leave, it is imperative to facilitate timely access to services aligned with the best evidence (8).

Primary care settings are typically the gateway to mental health care and services. In Canada, family physicians and specialized nurse practitioners (SNP) are responsible for prescribing and managing sick leaves related to CMD. This role entails coordination of referrals to appropriate health services and ensuring timely communication with the insurer and employer. In the Canadian province of Québec, to facilitate close interprofessional collaboration, family physicians and SNPs often work in family medicine groups (FMG) alongside a variety of other professionals. However, growing literature supports that family physicians feel ill-equipped and lack the time to take on this role, which influences the care pathways of patients with CMD and contributes to delaying access to the required psychosocial and work rehabilitation services (18, 21–23).

Several studies have demonstrated that RTW coordinators play a pivotal role in improving recovery, and timely and sustainable RTW (16, 17, 24). With knowledge of existing services and organizational procedures, the RTW coordinator’s role is centered on ensuring collaboration and mutual understanding of each stakeholder’s role and expectations as well as facilitating coordinated actions among them. Accordingly, they provide support to the worker in identifying their needs in terms of services to ensure recovery and sustainable RTW (25). In Quebec, RTW coordinators are often employed by employers or insurers and can be seen by workers as being in a position of conflict of interest, which can limit the impact of their interventions and the trust that workers on sick leave have in their interventions (16, 18). To offer neutral support, it is recommended that RTW coordinators should be in an independent position within the healthcare system (26). However, only a few studies have evaluated the implementation of this type of intervention in a primary care setting for people with CMD. One of these studies is being conducted in Germany and aims to demonstrate the effectiveness of an interprofessional team program that focuses on co-orientation, coordination, and cooperation to promote sustainable RTW (27). Three intervention modules are offered to support patients and to act on RTW facilitators and obstacles, develop work capacities, and prevent and manage relapses. Another set of studies evaluated different components and perspectives of the mandatory integration of RTW coordinator in the Swedish primary healthcare system since 2020 (28–30). Results published until now describe facilitators and obstacles to the coordination of RTW in primary care and to the collaboration of RTW coordinator with family physicians and employers (28, 30, 31). Facilitators identified include achieving open dialogue and common goals between primary care workers and insurer and employer representatives, establishing common value systems towards intersectoral collaboration within the primary care team and ensuring collegiality between RTW coordinators (31). Barriers identified include variability in workplace resources and RTW conditions, unclear definition of the coordination role and lack of coordination training (31). They also describe that the RTW coordinator’s intervention in primary care provides the worker, the family physician, and the employer with insight on how to address difficulties in supporting the person’s recovery and RTW process (28, 30).

Since RTW coordinator's skills include assessing the person's capacities and identifying the daily and work tasks that suit these capacities, previous research has demonstrated that occupational therapists are one of the best-equipped professionals to intervene as RTW coordinator (16). Their training ensures that they acquire the competencies and skills necessary for coordinating and providing RTW interventions for people with CMD (26, 29, 32). In the context of sick leave, occupational therapists have the skills to provide interventions to support recovery, assess and improve the functional capacities and modifications to the work environment, such as adequate work accommodations, to support, in collaboration with the employer, sustainable RTW (33). However, recent Canadian studies on the integration of occupational therapists into primary care found that referrals to occupational therapy services are still uncommon, largely due to limited understanding of their role by primary care physicians and professionals, and because of various contextual barriers (34, 35). Moreover, while the number of studies on occupational therapists’ role in primary care has been increasing, there remain few that study the interventions directly in a specific primary care clinical setting (35).

### 1.1 Study objectives

In this context, the overall aim of this study is to evaluate the acceptability and explore the effects of an occupational therapist-led program, integrated within FMG in the Canadian province of Québec, that is designed to improve the management of CMD-related sick leave and promote service workers’ recovery and sustainable RTW.

We will describe the perspectives of 1) patients receiving the intervention, 2) occupational therapists providing it, and 3) FMG teams collaborating on its delivery on the acceptability of implementing an occupational therapy-led program in FMG. The effect this program has on the recovery and RTW outcomes of people with a CMD will be evaluated.

## 2 Methods and analysis

### 2.1 Conceptual frameworks

Two conceptual frameworks will be used in this study. The first one is the Theoretical Framework of Acceptability (TFA) developed by Sekhon and collaborators (36). This framework defines acceptability in terms of seven components described in **Table 1**. It helps to identify the barriers perceived by patients that can limit the implementation of an intervention in a given context. The TFA describes that acceptability can be assessed at three time points: 1) prospective acceptability, which allows for assessment of anticipated acceptability; 2) concurrent acceptability, which is assessed whilst participating in the intervention; and 3) retrospective acceptability, which is the assessment of experienced acceptability. In the current study, acceptability will therefore be assessed at three times: 1) before the implementation of the intervention (pre-implementation) to identify factors likely to influence implementation and acceptability (*prospective acceptability*); 2) during the intervention, to identify these factors as they arise (*concurrent acceptability*); and 3) after the intervention is implemented (post-implementation), to document the experience patients had of the intervention (*retrospective acceptability*) (36).

**Table 1:** Components of the Theoretical Framework of Acceptability (TFA) (1).

Components	Definitions
Affective Attitudes	Feelings of the person regarding the intervention.
Burden	The amount of effort perceived to be needed to engage in the intervention.
Ethicality	The degree to which the intervention aligns with a person’s value system.
Intervention coherence	The level of understanding of the person regarding the interventions and its functioning.
Opportunity Costs	The degree to which benefits, profits, or values are forfeited to engage in the intervention.
Perceived Effectiveness	The perceived potential of the intervention to meet its goals.
Self-efficacy	The person’s belief in their capability to carry out the actions needed for the intervention.

The second framework is the revised version of the Consolidated Framework for Implementation Research (CFIR) (37). The CFIR has been frequently used in implementation research to document contextual facilitators and barriers influencing the implementation of innovations (37). It includes 48 constructs and 19 subconstructs grouped into five domains (i.e., innovation, outer setting, inner setting, individuals, and implementation process). It will be used in this study to document elements of the context (other than those related to the intervention domain included in the TFA) that could influence the implementation of the occupational therapy program and will guide the development of the interview guides. For example, these elements could be related to the inner setting domain such as work organization of tasks, relational connections, communication, tension for change or relative priority for change. They could also be related to the individual domain (e.g., knowledge, skills, leadership and motivation of members of the healthcare team) and the implementation process domain (e.g., teamwork between all stakeholders involved, planning of roles and responsibilities, implementation strategies used).

### 2.2 Study design

A mixed methods multiple case study research design will be used (38). Case study is a naturalistic research approach that allows to explain and explore a phenomenon in its context (38), in this case, the implementation of an occupational therapy program in a new environment that is the FMG. This research approach will provide an in-depth understanding of the perspective of those who deliver the intervention in an FMG context, as well as those who receive it. It will also document the elements of the intervention and the context on which action must be taken to optimize its effects (39).

To achieve an in-depth understanding of the acceptability and explore the effects of each case, the present study will use a mixed methods case study design with a convergent design (40). For the acceptability assessment objective, the quantitative and qualitative components of this study will document the evolution of acceptability from the pre-implementation phase to the post-implementation phase and evaluate the similarities and differences between the perceptions of the various stakeholders, including health care professionals, insurers, employers, union representative.

To explore the effects of the occupational therapy program, a pre-test/post-test research design without a control group will be used. Quantitative data will be collected to measure the changes produced by the intervention on the patient’s recovery and RTW outcomes. The qualitative component will explore the patient’s perceived outcomes of the program in more depth.

This study is also rooted in a participatory research and integrated knowledge translation approach, which includes collaborators and important actors as members of the research team (41, 42). Our research team is composed of researchers, patient partners and occupational therapists. Partnerships with the clinical settings and managers were established at the start of the study planning to ensure the objectives of the study met the needs of their patients and of the clinical teams. They were involved in the development of the protocol and the data collection procedures. Their planned implication in each stage of the study has been documented throughout the methodology below. Furthermore, for each case, an implementation support committee, composed of members of the research team, of members of the FMG, and of patient partners will also be set up to support key project decisions, the interpretation of study findings, and participate in the knowledge mobilization activities. They will meet three to four times a year, as required, throughout the study period.

### 2.3 Case descriptions

The cases in this study are three university family medicine groups (U-FMG) recruited on a voluntary basis. In Québec, U-FMGs are family medicine groups that have an added academic component requiring implication in research activities and training of residents in family medicine, students, and interns in various disciplines. They are typically composed of family physicians, SNPs, nurses, social workers, pharmacists, and allied health professionals chosen by the clinic managers. Therefore, each case includes: 1) the patients, 2) the U-FMG teams (i.e., family physicians, SNPs, allied health professionals and clinic managers), and 3) the occupational therapists who will deliver the intervention.

The three U-FMGs are each in a different urban area and one of them also serves a rural population, allowing us to explore the influence of different factors such as access to psychosocial work rehabilitation services, which may be more limited in rural areas. Regional variability can allow diversity in the types of jobs held by patients and work settings, which may influence the RTW and recovery process and outcomes (43).

### 2.4 Occupational therapists and U-FMG team members trainings

Each U-FMG will have a full-time occupational therapist. During the pre-implementation phase, they will follow a continuing education program to make sure they provide services based on the most recent research evidence. This training will serve to refresh and update knowledge and skills on the principles of the recovery-oriented approach and the therapeutic RTW approach for CMD (44). The content of the pre-implementation continuing education program that will be offered to the occupational therapists is presented in **Supplementary Table 1**.

The occupational therapists will be mentored by two expert occupational therapists currently providing work rehabilitation interventions for people with CMD and continuing education in work rehabilitation for occupational therapists. They will also benefit from exchange platforms with each other to promote collegiality. These conditions have been put in place to provide sufficient support to these occupational therapists while they integrate independently a new practice context that has no or limited experience in collaborating with occupational therapists and RTW coordination.

Before the recruitment of participants, the three participating U-FMG team members will also receive training on the role of occupational therapists for patients with CMDs and on RTW coordinator. The training will last two hours. It will clarify patient referral criteria and processes, the services the occupational therapist can offer, the shared roles between health care professionals, and the collaboration and communication tools to be used between team members (e.g., referral form, use of electronic medical record, insurance forms). It will also promote the use of a common recovery perspective among all members of the U-FMG team. Previous experiences integrating a new professional into FMGs demonstrated the importance of understanding and communicating one's role to collaborate effectively with teams (34, 45, 46).

### 2.5 Description of the intervention

The occupational therapy program consists of three components based on the best current recommendations for the management of workers with CMD, namely 1) a consultation/prevention component, 2) a recovery and RTW coordination services component, and 3) a support in recovery and work rehabilitation component (11, 15, 33, 47). As planned in the program, family physicians will be asked to refer their patients to the occupational therapist in two distinct situations: 1) if they are questioning the appropriateness of prescribing a CMD-related sick leave, or 2) if they have prescribed sick leave. Occupational therapists will then determine which components of the program they will offer to the referred patients according to their situation and their needs (i.e. characteristics of their condition, insurance coverage, and work situation). A description of the intervention according to the TIDieR checklist is presented in **Table 2** (48).

**Table 2:** Description of the occupational therapy program based on TIDieR *(* 48).

Item	Description
Brief name	Primary care-based occupational therapy program for recovery and RTW – CMD
**WHY (rationale of intervention)**	1. Support family physician or SNP decision-making process concerning sick leave and RTW, 2. Coordinate the services required by the patient within the U-FMG and externally, 3. Facilitate patient recovery and RTW.
**WHAT (material & procedures)**	Component 1 - Consultation/prevention - Assessment of the impact of CMD on the patient’s daily life and work capacities. - Identification of factors contributing to the patient’s functional disabilities. - Support clinical decision-making process of family physicians and SNPs regarding sick leave and RTW (33, 44). - Recommendations to the patient and the employer to adapt work tasks or modify characteristics of the work environment (33). Component 2 - Coordination of recovery and RTW services - Coordination of services required to promote the patient’s recovery and RTW (RTW coordinator’s role) (25, 44). - Assessment of the patient’s needs and resources (e.g. insurance coverage) - Referral to the right professionals. - Communication, with the patient’s consent, with other professionals, the insurer, and the employer to foster concerted actions between stakeholders. RTW. Component 3 - Support in recovery and work rehabilitation *If the patient does not have access to work rehabilitation services outside of the U-FMG.* Offer interventions as described in the Therapeutic RTW program, including one or multiple of these phases: - Diagnosis of work disability: Using comprehensive assessment tools to assess the person’s current situation (e.g., *Work Disability Diagnosis Interview* (WoDDI)). - Preparation to work phase: improvement of quality of life and work prerequisite. - RTW phase: progressive development of work capacities and work adaptations in the real work environment. - Maintenance at work phase: assist the person in transferring knowledge in the real work environment and offer RTW support.
**WHO PROVIDED (intervention provider and training)**	Occupational therapist working within the U-FMG in collaboration with an interdisciplinary team and stakeholders. This occupational therapist will have received the training described in **Supplementary Material 1**. Family physicians and SNPs of the U-FMG referring patients to the occupational therapist.
**HOW (modes of delivery)**	The occupational therapist intervention is delivered using a recovery-oriented approach and is tailored to the patient's preferences and needs, as well as to the needs of the intervention.
**WHERE (infrastructure and relevant feature)**	Interventions can be provided by teleconsultation or in-person at the clinic, or the workplace, depending on the patients' needs and preferences.
**WHEN AND HOW MUCH (number of sessions, duration, intensity)**	Patients will be referred to the occupational therapist as soon as possible to prevent sick leave or ensure appropriate RTW coordinator support and referrals to external resources. Interventions may vary depending on whether the patient has access or not to occupational therapy services in the private system. If occupational rehabilitation services are offered by the U-FMG, we expect a one-hour appointment per week for two to up-to 16 weeks, depending on the patient's health and needs, as well as the characteristics of his or her job and work environment.
**TAILORING (personnalization)**	The intervention will be tailored to the patients’ needs and insurance coverage. It may therefore vary in terms of intensity of follow-up and duration. Patients will be referred to private services when they have access to them, and the occupational therapist will act primarily as a RTW coordinator in this situation. For patients who do not have access to private services, the intensity of interventions will be greater to provide psychosocial support and work rehabilitation services RTW.
**HOW WELL (planned)**	Occupational therapists will receive training before the study begins and mentorship will be offered to ensure that the interventions offered are delivered in respect of the recovery-oriented approach and the therapeutic RTW approach for CMD. U-FMG teams will also receive training on the role of the occupational therapist.

### 2.6 Patient recruitment strategy

For recruitment purposes, physicians and SNPs will be asked to identify patients with a diagnosis of CMD for whom a decision of sick leave has been made or may be considered. These patients will be referred to the occupational therapist. If they agree to participate in the study, a member of the research team will meet them to verify their eligibility, and to obtain their informed consent to participate in the study. Recruitment will take place over 12 months in each U-FMG.

To be eligible patients must be: 1) consulting a family physician or a SNP for a CMD, 2) referred to the occupational therapist for a new episode of sick leave or wish to prevent a sick leave, and 3) able to speak, read, and understand French or English. In this study, CMDs include depressive disorders, anxiety disorders, obsessive-compulsive disorder, and trauma- or stress-related disorders, including adjustment disorders (49). Patients will not be eligible if they: 1) have a diagnosis of a severe mental disorder limiting work integration or participation and 2) require or receive services from a specialized mental health care team.

### 2.7 Data collection

#### 2.7.1 U-FMG and participants' characteristics

##### 2.7.1.1 Case study characteristics

To provide a rich description of the study context, the following information on each U-FMG will be collected: number of registered patients, physicians, other professionals, and available resources (e.g., electronic medical record). Managers of the U-FMG will be consulted and local data will be used to collect this information. Qualitative data on the characteristics of the U-FMG's will also be collected in a focus group using a semi-structured interview guide based on the CFIR domains and constructs.

##### 2.7.1.2 Patients' sociodemographic characteristics and RTW related factors

Data will also be collected to describe the patients who received the occupational therapy program. An online questionnaire will be administered at the time of recruitment to document the following patient characteristics: gender, sex, age, race/ethnicity, education level, marital status, employment status, type of employment, household income, diagnosis, number of previous sick leave episodes and their duration, reasons for sick leave, type of insurance coverage, number of days absent from work, number of hours worked per week if partial sick leave, type and sector of employment, and company size. Source of referral (either the family physician or the SNP) will also be documented, whether the patient is or not registered with this professional, and since when.

#### 2.7.2 Characteristics of health care and social services received by patients

##### 2.7.2.1 Description of the occupational therapy interventions received

To document how occupational therapy interventions are implemented in the three U-FMGs, each occupational therapist will fill out a logbook for each patient describing frequency, duration, types, and modality of the interventions provided. Furthermore, information on stakeholders’ involvement, and on collaboration with other U-FMG team members, external professionals, and stakeholders will also be collected.

##### 2.7.2.2 Description of other health and social services used by patients

We will also document information on other health and social services accessed and used by the patient (e.g., psychologist, Employee and Family Assistance Program, private occupational therapy, etc.) via a self-administered questionnaire completed at the end of the study to evaluate the possible influence of accessing those resources.

#### 2.7.3 Assessing the acceptability of the occupational therapy program (objective 1)

Acceptability of the intervention will be assessed in three phases: pre-implementation, during implementation, and post-implementation (**Table 3**).

**Table 3:** Accessibility assessment data collection according to study frameworks and implementation phase.

Data collection methods	TFA	CFIR	Pre-implementation	During implementation	Post-implementation
TFA questionnaire *	X		X		X
Focus Groups**	X	X	X		X
Minutes from implementation support committee meetings**	X	X		X	
Research coordinator and occupational therapist logbook**	X	X		X	
Emails between research team and implementation support committee **	X	X		X	
Semi-structured interview with occupational therapists**	X	X			X
Semi-structured interview with patients**	X				X

*Quantitative data collection; **Qualitative data collection.

##### 2.7.3.1 Pre-implementation acceptability

Two strategies will be used to document pre-implementation acceptability: TFA questionnaire and a focus group in each participating U-FMG.

The TFA questionnaire will be completed by U-FMG team members, occupational therapists, and patients at the time of recruitment. It is an 8-item generic questionnaire assessing the seven components of acceptability. It can be adapted for any intervention (50). Each item is scored on a five-level Likert scale which allows to produce a score from 8 to 40, 40 being the highest level of acceptability. It was developed using participatory methods and is based on Sekhon et al. (36) theoretical framework. For the purpose of our study, it will be adapted to the context of the occupational therapy program with the support of our research team, which includes our patient partners, a family physician and occupational therapists. It will also be translated into French using online translation tools and validated through the consultation of our research team. Pretest of the questionnaire will be conducted with five patients, healthcare professionals and occupational therapists not participating in the study to ensure clarity of items.

The pre-implementation focus group will be conducted in each U-FMG to understand team members’ expectations related to the implementation of the program. The focus groups will involve 9 participants, including physicians and SNPs (n=4), U-FMG managers (n=3), a nurse (n=l), and a social worker (n=1). An interview guide based, on the TFA model by Sekhon et al. (36), will be used to document expectations, needs, readiness to change, perceived sense of control, and contextual elements that could facilitate or hinder the implementation of the intervention according to each of the model's domains. The group will be facilitated by a member of the research team and a patient partner. This data will be used to adapt the occupational therapy program according to the characteristics of the U-FMG context (e.g., team functioning, modes of communication, sharing of roles between team members).

##### 2.7.3.2 Acceptability during implementation

At each U-FMG, an implementation support committee will be set up to ensure effective coordination of the implementation and data collection processes. Meetings of the implementation support committee will be recorded, and minutes of each meeting will be produced. Moreover, elements facilitating or hindering the implementation of the occupational therapy program will be documented. The research coordinator will keep a logbook describing the steps taken each week (highlights from emails, phone calls, or meetings). Occupational therapists will also keep a logbook to document their perceptions of challenges or successes in implementing interventions and collaborating with other U-FMG professionals. E-mail exchanges between members of the research team and members of the implementation support committee will also be analyzed to document collaboration.

##### 2.7.3.3 Post-implementation acceptability

Three strategies will be used to document post-implementation acceptability: TFA questionnaire, semi-structured interviews, and focus groups.

The TFA questionnaire will be completed a second time, at the end of the implementation phase, by the U-FMG team members and the occupational therapists to compare pre- and post-implementation acceptability results. To document the perspective of physicians and SNPs who would not have referred patients to the occupational therapist, an additional open-ended question will be added to document the reasons associated with these practices. Patients who will have received the occupational therapy program will also complete the TFA questionnaire at the time of discharge in occupational therapy for post-implementation assessment.

Semi-structured interviews will be conducted with ten to 15 participating patients from each of the U-FMGs. A maximum variation purposeful sampling strategy will be used to recruit patients with different characteristics within the sample of subjects who received the intervention (51). We will recruit patients in consideration of their gender, age, level of education, socio-economic level and whether or not they had access to private insurance. Patients who had previously experienced a sick leave related to a CMD will also be recruited to compare their experiences in terms of access to services and perceived effects. The interview guide will be developed based on the TFA model. Semi-structured interviews are expected to last between 30 and 60 minutes and will be conducted via a web conference platform.

Interviews will also be conducted with the participating occupational therapists, using an interview guide based on the TFA model and the CFIR to document the occupational therapists’ perspective on the acceptability of their interventions and the contextual factors that influenced the acceptability of the program. They will also be asked to comment on the future, larger-scale implementation of the program in other U-FMGs or FMGs. Semi-structured interviews will last approximately 60 minutes and will also be conducted via a web conference platform.

We will also conduct a focus group with each of the U-FMG teams. The group will be composed of approximately nine participants, including physicians and SNPs (n=4), U-FMG managers (n=3), a nurse (n=l) and a social worker (n=1). An interview guide also based on the TFA model and CFIR framework will be used. The group will be facilitated by a member of the research team and a patient partner. **Table 3** summarizes the data collection methods planned at each phase for the accessibility assessment.

#### 2.7.4 Exploratory evaluation of the effects of the occupational therapy program (objective 2)

Data collection on the exploratory effects of the occupational therapy program on recovery and RTW will be carried out at baseline, at discharge, and at 6 months follow-up.

##### 2.7.4.1 Effects on recovery

To explore the effects of interventions on patient recovery, we will assess depressive and anxiety symptoms, perceived health-related quality of life, occupational balance and personal recovery using the following five questionnaires:

##### 2.7.4.2 Patient health questionnaire-9

This questionnaire will be used to assess the intensity of depressive symptoms (52). The PHQ-9 has adequate reliability, excellent discriminant and convergent validity, and a robust structure factor (52, 53).

##### 2.7.4.3 General anxiety disorder

This questionnaire will be used to measure the intensity of anxiety symptoms (54). The GAD-7 has excellent sensitivity, specificity, convergent validity and internal consistency, enabling it to be a good screening tool for anxiety symptoms (55, 56).

According to guidelines provided by the Quebec Ministry of Health and Social Services (57) for mental disorder, the GAD-7 and PHQ-9 should be administered when symptoms associated with frequent mental disorders in adults are present.

##### 2.7.4.4 Short-Form 12

The Short-Form 12 (SF-12) will be used to measure self-perceived health-related quality of life and includes eight domains assessing physical and mental health (58, 59). The SF-12 is an adapted version of the SF-36, whose validity, test-retest reliability and responsiveness to change have been repeatedly demonstrated in the literature (60, 61). This test has also been validated to assess health-related quality of life in cohorts with mental health conditions (62).

##### 2.7.4.5 Occupational Balance Questionnaire

The Occupational Balance Questionnaire will be used to document the person’s level of life balance, i.e., the extent to which a person achieves a good quantity and variation of occupations. The score of this questionnaire is strongly correlated with perceived health (63, 64). The original version demonstrates a good internal consistency, and satisfactory test-retest reliability (63). The French-language version of this questionnaire demonstrated good internal consistency and satisfactory test-retest reliability in Quebec (64).

##### 2.7.4.6 Brief INSPIRE-O

The Brief INSPIRE-O is an adapted version of the Brief INSPIRE designed to assess personal recovery outcomes. It is a five-item self-reported outcome measure based on the CHIME framework for recovery (65). Each item is meant to assess one of the five processes described by the CHIME framework: Connectedness, Hope and optimism, Identity, Meaning and purpose, and Empowerment (66, 67). Its validation demonstrated good scalability, satisfactory internal consistency, and moderate test-retest reliability (65).

##### 2.7.4.7 Effects on RTW

Effects on RTW will be assessed by documenting work status at discharge and at six months follow up using a validated grid (68), job type, and self-reported duration of absence from work. In addition, two questionnaires will be used to document perceived RTW self-efficacy and work functioning at baseline, at discharge and at six month follow up.

##### 2.7.4.8 RTW self-efficacy questionnaire

The RTW self-efficacy questionnaire (RTW-SE) will be used to evaluate RTW self-efficacy. The original English version of the RTW-SE has excellent internal reliability, adequate test-retest reliability and good sensitivity to change, and is identified in the literature as having predictive validity for RTW (69, 70). The French-language version was translated in a two-stage process by the Institut de recherche Robert-Sauvé en santé et sécurité du travail (71).

##### 2.7.4.9 Work Role Functioning Questionnaire

We will use the Work Role Functioning Questionnaire (WRFQ) to evaluate work functioning, the extent to which an induvial feels able to meet the physical, mental, and social demands of the job (72, 73) The Canadian French version of the WRFQ has good content validity, very good internal consistency for all items except the social demands scale, and good construct validity (72, 74).

### 2.8 Analysis

The Framework Method will be used to analyze the data associated with each objective. It is an analytical method suitable for multiple case studies, mixed research designs, and for managing large amounts of data (75). The seven steps of the method will be used for each case: 1) transcription of the data, 2) familiarization with the data, 3) coding, 4) development of an analytical framework, 5) application of the analytical framework, 6) classification of the data in the analytical matrix, and 7) interpretation of the data. All qualitative and quantitative data will be integrated into the NVivo 14 analysis software to create the results matrix. To contextualize the data for each case, the contextual data will be analyzed using the CFIR domains and constructs to create a portrait of each study site (37). The results of the analysis will be discussed with the research team members.

#### 2.8.1 Objective 1 - acceptability assessment

Data collected for pre-implementation, during implementation and post-implementation assessment will be analyzed separately using a combined deductive and inductive thematic analysis approach (75, 76). Data will be organized to document each of the TFA domains. Two members of the research team will iteratively cross-code the data, reviewing the analyses performed for each case on an alternating basis to improve the intersubjective traceability of the coding.

Quantitative data collected using the TFA questionnaire will be analyzed using descriptive statistics, and differences between pre- and post-implementation results will be assessed with a paired t-test (or Wilcoxon signed ranks test if data are not normally distributed) using SPSS 29 software.

The results of the quantitative and qualitative analyses of acceptability will be reported into a joint display table to compare them for each of the components of the TFA model and will consider the different levels of analysis, i.e. acceptability at the U-FMG level, for the different professionals and for the patients (77). The results matrices produced for each case will then be compared (cross-case analysis) to analyze the influence of context variables on levels of acceptability pre-, during and post-implementation of interventions (38).

#### 2.8.2 Objective 2 - exploratory evaluation of effects

Quantitative data on recovery and RTW will first be analyzed for each case independently, to document the effects produced by the intervention program by comparing the results obtained at baseline, discharge, and six months after the end of the interventions. Depending on the nature of the data, either parametric (ANOVA) or non-parametric (Friedman test) repeated measures statistics will be used. Analysis evaluating the differences between the results obtained for patients from the three U-FMGs will also be carried out using SPSS 29 software.

### 2.9 Trustworthiness

Strategies will be used by our team to ensure the trustworthiness of our findings. The study will allow the research team to have a prolonged engagement with each site helping them to achieve a nuanced insight of the phenomenon under study. A research journal will be kept by the research coordinator and the occupational therapists providing the intervention to document factors influencing the acceptability and implementation of the intervention. As described previously, we will use triangulation to collect various sources of data from multiple stakeholders. We will also thoroughly document the research process and decisions made by the research team and the implementation support committees of each research site. Debriefing with the research team will be performed regularly throughout the study. In-person group meetings will be conducted at the time of final data analysis for results validation. Results will also be presented at each research site to improve confirmability of findings. Transferability will be ensured by the thorough description of the research context, participants and also by collecting data on the perspectives of stakeholders in regard to the possible implementation of the program in other U-FMGs and FMGs in the province of Quebec.

## 3 Discussion

Over the past 20 years, evidence on how to best manage sick leaves associated to CMDs has been accumulating (2, 13–15, 44). Literature also describes the challenges preventing the implementation of these recommendations in practices in the Canadian context such as the lack of access to care and management from primary care providers (21, 22). The present study proposes a solution to put evidence on the management of sick leaves for patients' CMDs into practice and contribute to transforming primary care for the benefit of patients. Considering the complexity of mental health sick leave managements, the need to follow a thorough development process is undeniable. Following the Medical Research Council’s Framework for the development and evaluation of complex interventions (78), this protocol illustrates even if this type of intervention has been previously described in the literature, it has not yet been tested in the specific and unique context of primary care. In that situation, the next phase of the development of knowledge on this kind of intervention relies in understanding how it should be adapted and how it is acceptable for patients, professionals and managers in this new delivery context It will also allow our team to assess the feasibility of the research methodology to prepare the conduct of a future experimental study if results from this study are promising.

### 3.1 Potential challenges and strategies to mitigate the risk

Some challenges and limitations to this study must be acknowledged. First, it will be important to have the collaboration of the family physicians and SNPs to refer patients to the new occupational therapists integrating their team. It will also be important that they have a good understanding of the occupational therapist’s role. Training and close monitoring of these factors in the U-FMGs will be important. On the other hand, it is possible that the occupational therapist will receive too many referrals. Should this occur, a waiting list will be created. It is expected that the intervention will be delivered in a timely matter to the participants and problem-solving strategies will be used to identify how to improve appropriate referrals and avoid delays in treatment. The participatory research approach will support the engagement of U-FMG managers and professionals, patient partners and the research team in the problem-solving process. Furthermore, given that the U-FMGs (cases) were selected, in large part, due to their interest towards improving CMD care and services, the level of acceptability of those clinics could potentially be higher than those of other FMGs. The use of a mixed methods multiple case study will provide an in-depth understanding of these three cases from three different regions and a better understanding of how contextual factors influence the acceptability of this type of intervention Transferability may be limited since the project will be conducted in the province of Quebec. The results will be transferable to countries with healthcare coverage similar to Canada's, i.e. universal healthcare coverage. Given the exploratory nature of the intervention effects, the generalizability of this part of the results will be limited. Finally, while beyond the scope of this study, it will be relevant and important to assess the cost-effectiveness of this intervention to determine if scaling-up should be promoted and how it can support changes to insurance coverage and wider provision of public services.

## 4 Conclusion

This study will allow further clarification and promotion of the importance of integrating occupational therapists into primary care to enhance the quality of care and services offered to the mental health population. This exploratory study on the acceptability and effects is the first step towards designing and conducting a randomized controlled trial and an economic evaluation to test the effectiveness and cost-effectiveness of implementing the intervention in a primary care clinical setting.
