# Supplementary material for: Acceptability and exploratory effects of an occupational therapy intervention to improve recovery and return to work of workers with mental health disorders in primary care: a mixed methods study protocol
Source: Front Psychiatry. 2024 Nov 28;15:1441855. doi: 10.3389/fpsyt.2024.1441855 (PMC11635104; doi:10.3389/fpsyt.2024.1441855)
Supplement: Supplementary file 1 [file Table1.docx]

**Supplementary Material 1 - Content of the pre-implementation continuing education program for occupational therapists**

| **Topics of continuing education program** | **Description** |
| --- | --- |
| Theoretical approaches and models | - Recovery oriented model and CHIME approach principles - Work Disability Prevention Model and paradigm - Therapeutic RTW model |
| Assessment of occupational performance and work disability for people with CMD | - Assessment of CMD symptoms - Assessment of occupational performance and participation in activities of daily living including occupational balance - Assessment of work disability related factors - Assessment of work requirements and work environment - Assessment of work performance and participation |
| Coordination of recovery and RTW services | - Navigation support in the healthcare and social services system and work disability system - Understanding the role, issues and interests regarding the return to work of each stakeholder (including healthcare professional, insurer, employer, union) - Interest-based negotiation approach between the insurer and the employer - Interventions to improve collaboration between stakeholders. |
| Recovery interventions | - Reflective learning surrounding recovery - Self-management approach - Empowerment and self-advocacy practices - Relevant use of community resources |
| RTW interventions | - Management principles related to quality of life, stress management, problem-solving skills, self-efficacy, communication skills - Development of the worker’s work capacities thru a physical/cognitive reactivation program - Preparation of a work environment that offers worker a therapeutic margin of maneuver. |
